# Bidirectional associations between physical activity and sleep in older adults: a multilevel analysis using polysomnography

**DOI:** 10.1038/s41598-022-19841-x

**Published:** 2022-09-13

**Authors:** Jaehoon Seol, Jaehee Lee, Insung Park, Kumpei Tokuyama, Shoji Fukusumi, Toshio Kokubo, Masashi Yanagisawa, Tomohiro Okura

**Affiliations:** 1 Faculty of Health and Sport Sciences, University of Tsukuba, Tsukuba, Japan; 2 International Institute for Integrative Sleep Medicine, University of Tsukuba, 1-2 Kasuga, Tsukuba, Ibaraki 305-8550 Japan; 3 Japan Society for the Promotion of Science, Tokyo, Japan; 4 Doctoral Program in Physical Education, Health and Sports Sciences, Graduate School of Comprehensive Human Sciences, University of Tsukuba, Tsukuba, Japan; 5 R&D Center for Frontiers of MIRAI in Policy and Technology, University of Tsukuba, Tsukuba, Japan; 6 Life Science Center for Survival Dynamics (TARA), University of Tsukuba, Tsukuba, Japan; 7 Department of Molecular Genetics, University of Texas Southwestern Medical Center, Dallas, TX USA; 8 R&D Center for Tailor-Made QOL, University of Tsukuba, Tsukuba, Japan

**Keywords:** Geriatrics, Public health, Quality of life

## Abstract

Although recent studies have examined the bidirectional associations between physical activity and sleep parameters, few have focused on older adults utilizing objective assessments, such as polysomnography. This micro-longitudinal observational study included 92 Japanese older adults (aged 65–86 years) who underwent objective evaluations of sleep quality using polysomnography and completed subjective sleep-related questionnaires. Activity levels were assessed using an accelerometer. Polysomnography, subjective sleep-related questionnaires, and accelerometer were administered for 7 consecutive days. Multilevel models (participant-, day-level) were used to examine the temporal associations of objective and subjective sleep parameters with sedentary behavior and physical activity. In the day-level analysis, higher levels of sedentary behavior during daytime were associated with longer rapid eye movement (REM) sleep, shorter REM latency, lower levels of non-REM sleep (stage N3), and reduced delta power during daytime. Higher levels of low-intensity physical activity during daytime were associated with lower levels of REM sleep, longer REM latency, and increased stage N3 sleep in the day-level analysis. Higher levels of moderate-to-vigorous physical activity were associated with increased REM latency. Longer subjective sleep time was associated with increased next-day moderate-to-vigorous physical activity. Thus, low-intensity physical activity may provide objective benefits related to deep sleep parameters in older adults.

## Introduction

Non-restorative sleep and difficulties initiating or maintaining sleep have been reported in approximately 50% of older adults^1^. Several studies have demonstrated that these disturbances are caused by age-related factors, such as decreased physical activity, social isolation, increased levels of depressive symptoms, and circadian rhythm alterations^2–5^.

The prevalence of insomnia is lower among older adults who engage in daily physical activity^6^, which exerts distinct effects on sleep quality depending on its intensity (e.g., sedentary behavior, low- or moderate-to-vigorous intensity)^7^; in particular, daily low-intensity physical activity (e.g., light walking, stretching, lifting hand weights, etc.) is associated with improved sleep quality^7,8^. Moreover, engaging in moderate-to-vigorous intensity aerobic exercise (e.g., cycling, running, etc.) also improves sleep quality^9,10^. Although the effects of exercise on sleep quality vary in magnitude depending on the type of activity^11^, daily physical activities, such as housework and work-related activities, are also positively associated with sleep quality in older adults^12^. Physical activity affects sleep quality by several mechanisms, including energy conservation, increases in body and central nervous system temperature, and decreases in anxiety^13–15^.

Several methods can be used to assess sleep quality, including validated questionnaires, wrist actigraphy, and polysomnography^5^. However, previous studies have reported low correlations between objective and subjective assessments of sleep quality^16,17^ as the two measures appear to reflect different aspects of sleep quality^17^. Subjective sleep quality is mainly measured via qualitative evaluation of the monthly average sleep quality (e.g., using the Pittsburgh Sleep Quality Index). In contrast, objective sleep quality is measured by actigraphy or polysomnography. Moreover, polysomnography can provide more detailed information on different sleep stages (e.g., shallow or deep sleep) in addition to other objective sleep parameters (e.g., total sleep time and sleep latency). Sleep architecture is related to collapsed circadian rhythm and the presence of depression, dementia, and mortality^18,19^. For example, rapid eye movement (REM) sleep is thought to reset the brain and has been associated with improvement in cognitive function the next day^18^. Specifically, while cholinergic neurons are important modulators of REM sleep, REM sleep itself has a positive recovery effect on cholinergic network impairments and on the imbalance between cholinergic and orexinergic systems^18^. Furthermore, a shorter REM sleep duration has been associated with higher mortality among older adults^19^.

Actigraphy can be used to assess sleep habits in daily life. Recent epidemiological studies have objectively examined the relationship between physical activity and sleep using accelerometer and actigraphy data^7,20,21^. Thirty minutes of sedentary behavior per day with an equal period of low-intensity physical activity was associated with decreased wakefulness after sleep onset and greater sleep efficiency among older adults^7^.

Technological developments in assessment methods have led to the development of various statistical methods, such as multilevel analysis. Continuous physical activity and sleep data are structured based on each participant, and multilevel analysis allows one to assess bidirectional relationships both between (level 2: participant-level) and within participants (level 1: day-level)^22^. Multilevel analysis can therefore be used to examine temporal directionality, both between and within participants, between sleep and physical activity throughout the week. This approach provides insight into the temporal directionality between physical activity and sleep and the mutual correlation after considering between-participant differences^22^. However, a common limitation among these studies is that they could not assess relationships based on sleep stage^23–26^.

Some previous studies have attempted to achieve more accurate quantitative and qualitative evaluations based on estimated sleep stages and delta power (0.75–4.00 Hz)^27,28^. Delta power refers to the scalp surface representation of a highly complex ensemble of oscillatory activity during non-REM sleep, including the cortical up and down states that comprise the slow oscillation^29,30^.

Actigraphy allows for quantitative evaluation but cannot provide qualitative data concerning sleep stage or delta power. However, polysomnography allows for both quantitative (e.g., total sleep time and sleep latency) and qualitative evaluations (e.g., shallow or deep sleep). To date, previous studies have not used continuous polysomnography due to the burden of unfamiliar sleep environments (e.g., laboratory and/or medical facilities) in older adults. In addition, skilled staff are required to attach polysomnography electrodes. Therefore, consecutive daily measurements using ordinary polysomnography are almost impossible, especially for older adults. To overcome these limitations, some studies have used simple home-based polysomnography devices under participants’ free-living home conditions^31,32^.

Several studies have demonstrated a bidirectional relationship between physical activity and sleep quality as measured using actigraphy or recorded using sleep diaries^22,23,26,33^. However, few such studies have utilized polysomnography, and the day-to-day bidirectional relationship between daily physical activity and objectively measured sleep depth (e.g., sleep stage) remains unknown. Based on previous evidence^7,8^, we hypothesized that increased day-to-day physical activity performed at low intensity would exhibit a linear relationship with objective parameters that are positively related to deep sleep (e.g., sleep stage 3 and delta power) and with REM sleep parameters. To evaluate our hypothesis, we examined the bidirectional associations between the objectively measured day-to-day intensity of physical activities and both objective and subjective sleep quality in older adults.

## Results

### Participant characteristics

We analyzed accelerometer and polysomnography data for 92 participants. The average number of days with valid actigraphy and polysomnography data was 6.5 ± 0.9 days. Further, the number of days for the self-report questionnaire (OSA-MA version) was 6.7 ± 1.0 days.

**Figure 1:**
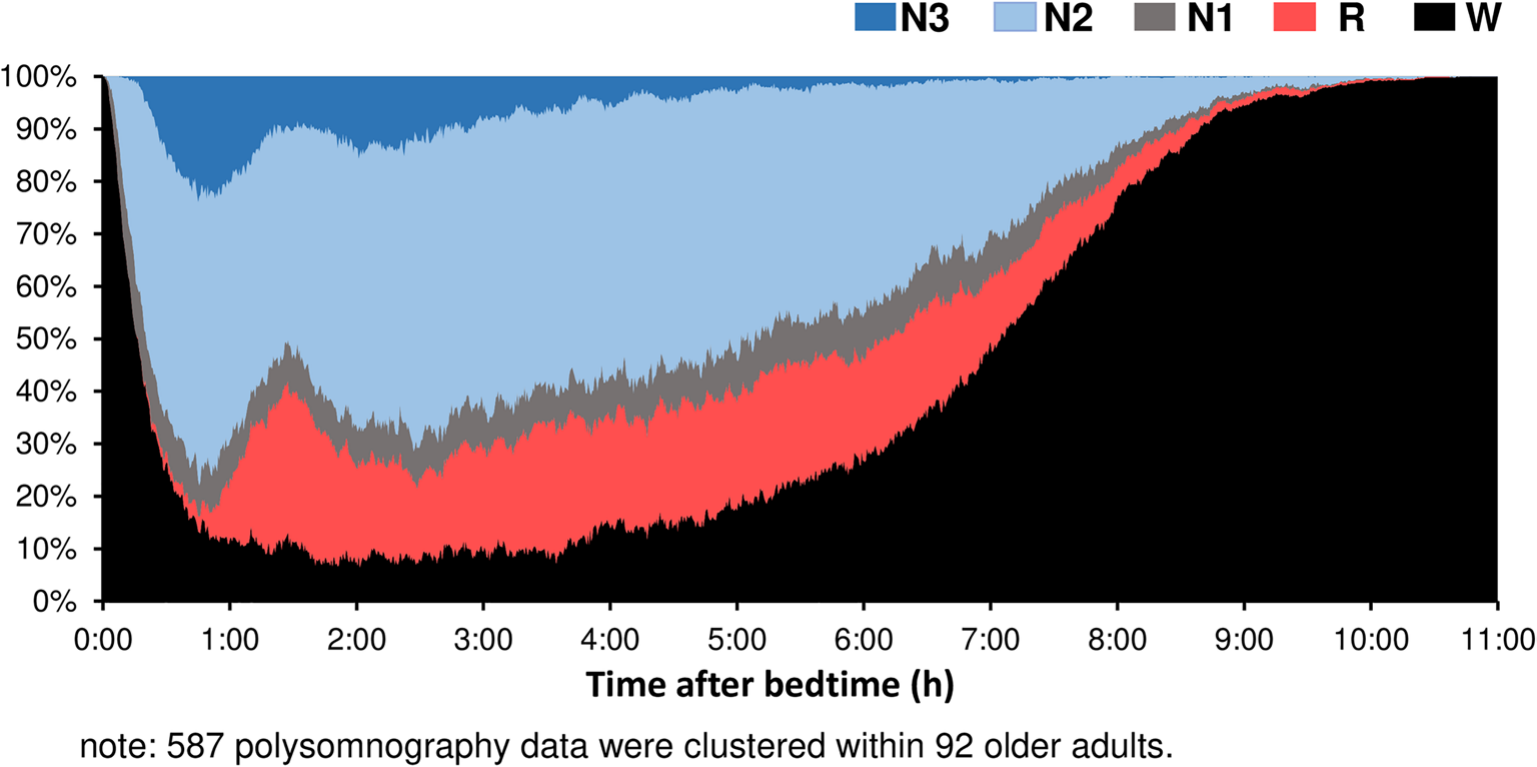
Cumulative display of sleep architecture in all 92 participants. The percentage of participants in each sleep stage is shown for stages W (black), N1 (gray), N2 (light blue), N3 (blue), and R (red). *W* awake, *R* rapid eye movement sleep, *N* non-rapid eye movement sleep. A total of 587 polysomnography data points were clustered in 92 older adults.

Participant characteristics are presented in Table 1, and the cumulative display of sleep architecture for all 92 participants is shown in Fig. 1. Time in bed and total sleep time among all participants were 7.7 and 6.2 h per day, respectively. The durations of daily sedentary behavior, low-intensity physical activities, and moderate-to-vigorous-intensity physical activities were 533.4 min per day (58.2% of wear time), 316.7 min per day (34.7% of wear time), and 64.6 min per day (7.1% of wear time), respectively (Table 1). We confirmed the intercorrelations among polysomnography and the OSA-MA version parameters. The sleep onset latency measured by polysomnography was significantly negatively related with sleepiness on rising (*r* = − 0.218), initiation and maintenance of sleep (*r* = − 0.277), and the OSA total score (*r* = − 0.255). The REM latency was significantly negatively related with initiation and maintenance of sleep (*r* = − 0.357). Additionally, the total sleep time was negatively related with frequent dreaming (*r* = − 0.217) and positively related with sleep length (*r* = 0.244) (all *P* values < 0.05).

**Table 1:** Characteristics of participants.

	Mean ± SD	Min	Max
Age, years	73.9 ± 5.0	65	86
Female, n (%)	73 (79.3)		
Body mass index, kg/m^2^	23.7 ± 3.2	16.9	38.3
Smoking history, n (%)	1 (1.1)		
Alcohol consumption (drinker), n (%)	35 (38.0)		
Caffeine consumption (drinker), n (%)	84 (91.3)		
**Medical history**			
Hypertension, n (%)	35 (38.0)		
Hyperlipidemia, n (%)	18 (19.6)		
Diabetes, n (%)	4 (4.3)		
GDS score, points	3.2 ± 2.8	0	14
**Objective sleep parameters**			
Time in bed, min	459 ± 62.3	243.6	612.1
Total sleep time, min	373.6 ± 44.6	208.9	452.9
Sleep latency, min	19.7 ± 11.0	4.3	60.6
WASO, min	68.1 ± 30.3	21.2	149.8
Sleep efficiency, %	81.4 ± 6.2	64.8	91.7
REM latency, min	96.7 ± 34.3	48.4	240.8
N1, %	10.5 ± 4.2	2.6	25.3
N2, %	58.3 ± 7.0	43.0	75.6
N3, %	7.9 ± 7.3	0	27.8
REM, %	23.3 ± 4.1	13.3	33.1
Sum of delta power, μV^2^/night	115,306 ± 9223	25,768	272,428
**Subjective sleep parameters**			
Sleepiness on rising, pts	51.8 ± 7.5	30.1	65.0
Initiation and maintenance of sleep, pts	46.8 ± 7.4	19.4	62.5
Frequent dreaming, pts	52.5 ± 6.2	38.8	58.4
Refreshing, pts	53.2 ± 7.0	37.4	65.6
Sleep length, pts	48.3 ± 6.7	28.8	63.5
OSA-MA total score, pts	252.6 ± 25.4	197.4	303.8
**Habitual physical activity**			
Wear time, hour	15.2 ± 1.4	11.8	18.3
SB, hour (% of wear time)	8.9 (58.2)	5.0	12.7
LPA, hour (% of wear time)	5.3 (34.7)	2.1	7.4
MVPA, hour (% of wear time)	1.1 (7.1)	0.1	3.5

*SD* standard deviation, *GDS* Geriatric Depression Scale (Sheikh JI and Yesavage, 1986), *WASO* wake after sleep onset, *N* non-REM sleep stage, *REM* rapid eye movement, *SB* sedentary behavior, *LPA* low-intensity physical activity, *MVPA* moderate-vigorous-intensity physical activity. n = 92.

### Temporal associations between daytime physical activity and predicted sleep quality that night

**Figure 2:**
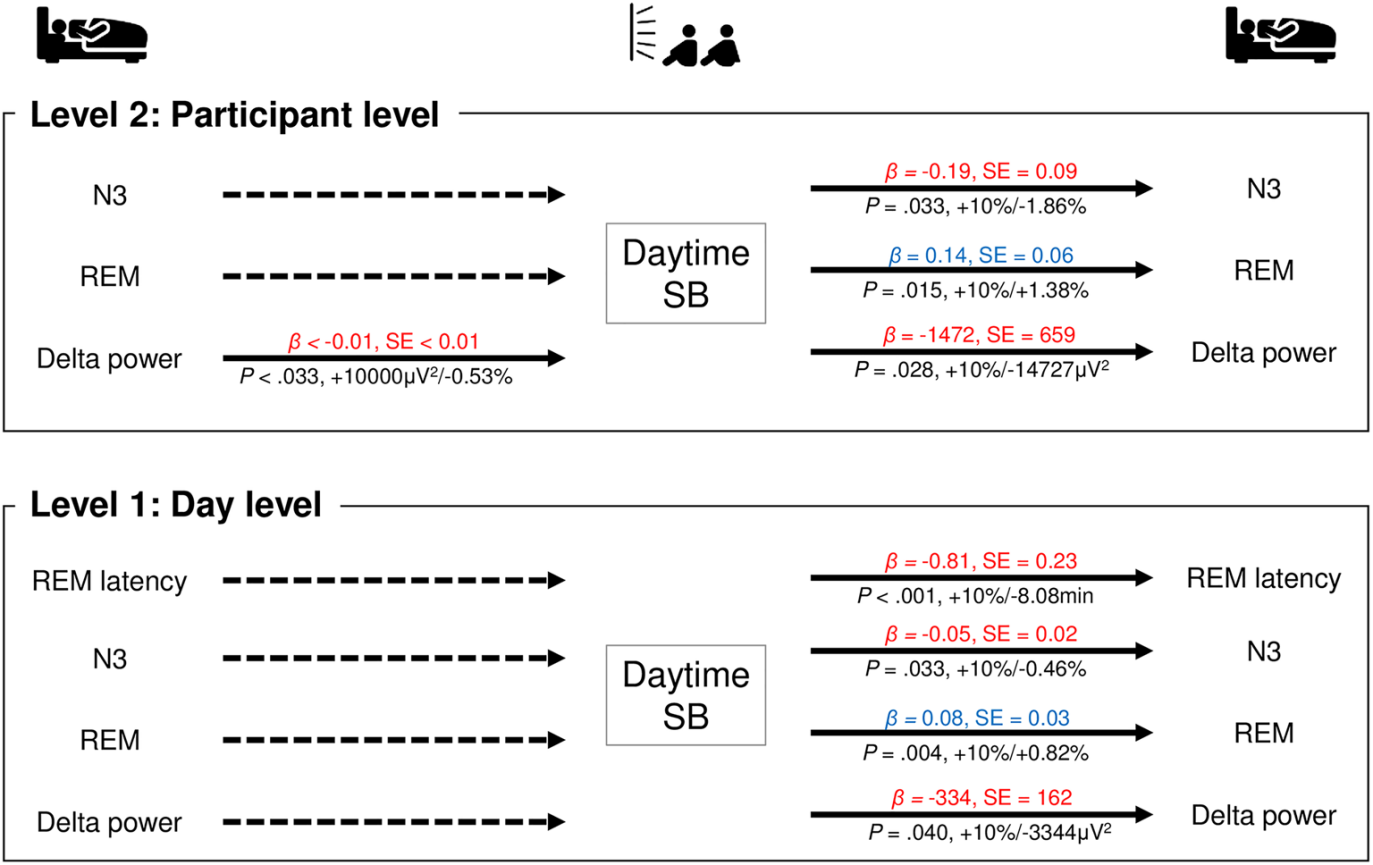
Bidirectional associations between sleep parameters and sedentary behavior. Adjusted for age; sex; BMI; history of smoking, alcohol consumption, and caffeine consumption; and medical history of hypertension, hyperlipidemia, and depressive symptoms. *SB* sedentary behavior; the solid line represents significant relevance. A solid line on both sides indicates bidirectionally related parameters, and a solid line on one side and a dashed line on the other side indicate only temporally related parameters. Blue text indicates a positive relationship, and red text indicates a negative relationship.

Same-day daytime sedentary behavior exhibited significant negative associations with REM latency (B = − 0.81, *P* < 0.001), stage N3 sleep (B = − 0.05, *P* = 0.033), and delta power (B = − 334.4, *P* = 0.040). Same-day daytime sedentary behavior also exhibited a significant positive association with REM sleep (B = 0.08, *P* = 0.004). As in the day-level analysis, analyses at the participant level indicated that daytime sedentary behavior was significantly and negatively associated with stage N3 sleep (B = − 0.19, *P* = 0.033) and delta power (B = − 1472.7, *P* = 0.028) that night. Daytime sedentary behavior also exhibited a significant positive association with REM sleep in the participant-level analysis (B = 0.14, *P* = 0.015) (Fig. 2, Supplementary Tables S1 and S2).

**Figure 3:**
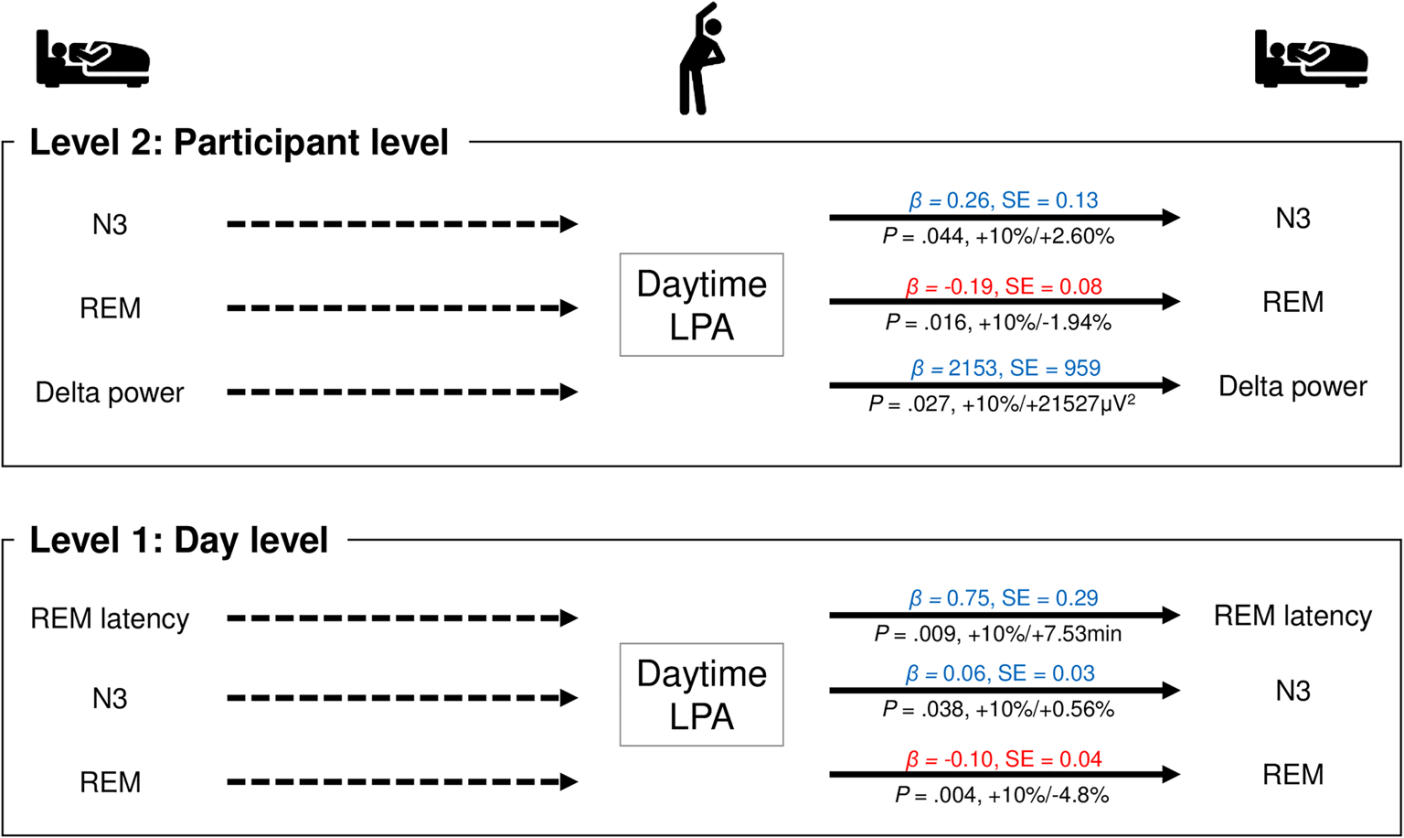
Bidirectional associations between sleep parameters and low-intensity physical activity. Adjusted for age; sex; BMI; history of smoking, alcohol consumption, and caffeine consumption; and medical history of hypertension, hyperlipidemia, and depressive symptoms. *LPA* low-intensity physical activity. The solid line represents significant relevance. A solid line on both sides indicates bidirectionally related parameters, and a solid line on one side and a dashed line on the other side indicate only temporally related parameters. Blue text indicates a positive relationship, and red text indicates a negative relationship.

Same-day daytime low-intensity physical activity exhibited a significant positive association with nighttime REM latency (B = 0.75, *P* = 0.009) and stage N3 sleep (B = 0.06, *P* = 0.038), as well as a significant negative association with REM sleep (B = − 0.10, *P* = 0.004). In the participant-level analysis, daytime low-intensity physical activity exhibited a significant positive association with stage N3 sleep (B = 0.26, *P* = 0.044) and delta power (B = 2152.7, *P* = 0.027) at night, as well as a significant negative association with REM sleep (B = − 0.19, *P* = 0.016) (Fig. 3, Supplementary Tables S3 and S4).

**Figure 4:**
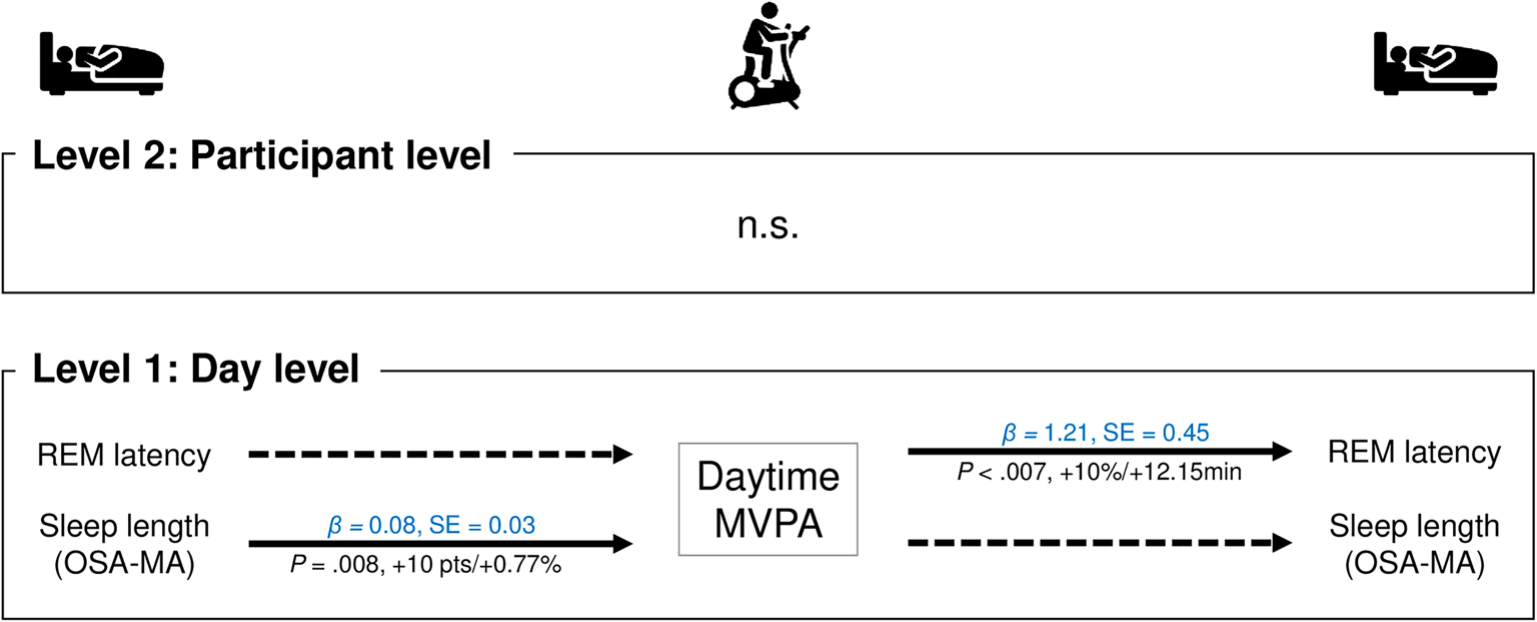
Bidirectional associations between sleep parameters and moderate-to-vigorous-intensity physical activity. Adjusted for age; sex; BMI; history of smoking, alcohol consumption, and caffeine consumption; and medical history of hypertension, hyperlipidemia, and depressive symptoms. MVPA, moderate-to-vigorous-intensity physical activity. The solid line represents significant relevance. A solid line on both sides means that they are bidirectionally related parameters, and a solid line on one side and a dashed line on the other side means that they are only temporally related to each parameter. Blue text means a positive relationship, and red text means a negative relationship.

Same-day daytime moderate-to-vigorous-intensity physical activity was significantly and positively associated with nighttime REM latency (B = 1.21, *P* = 0.007). However, there were no significant associations of daytime moderate-to-vigorous-intensity physical activity at the participant level (Fig. 4, Supplementary Tables S5 and S6).

### Temporal associations between nighttime sleep and predicted physical activity the next day

The participant-level delta power was significantly and negatively associated with daytime sedentary behavior (B < − 0.01, *P* = 0.033) (Fig. 2, Supplementary Table S7). Additionally, same-day subjective sleep duration was significantly and positively associated with daytime moderate-to-vigorous-intensity physical activity (B = 0.08, *P* = 0.008) (Fig. 4, Supplementary Table S8). However, there were no significant relationships between next-day physical activity and other sleep parameters (Figs. 2, 3, 4, Supplementary Tables S9–S21).

## Discussion

In the present study, we examined the bidirectional associations between physical activity and both objective and subjective sleep quality in older adults. Our analysis revealed that the characteristics of stage N3 sleep were better among our participants than among those included in a previous epidemiological study^28^. In 80% of younger adults, changes in sleep structure related to stage N3 appear within approximately 1 h after falling asleep^34^; however, such changes occurred in only 20% of older adults in the current study (Fig. 1). Compared to American, Swiss, and Brazilian older adults, the Japanese participants showed short total time in bed; however, a similar sleep structure has been reported in older adults from other countries^19,35,36^. Overall, compared with the National Sleep Foundation’s sleep quality recommendations^37^, the participants seemed to be relatively healthy in terms of sleep composition.

Previous studies have focused on the bidirectional association between physical activity and sleep; however, these studies primarily assessed physical activity and/or sleep using subjective measures^33,38,39^, and most quantitative evaluations of sleep have been performed using actigraphy only^22,23,26^. These studies suggested qualitative assessment of sleep as a direction for future research^22,24,40^ and commonly mentioned that future studies should use polysomnography data for both qualitative and quantitative estimation^24,33,41^. Moreover, few studies have focused on the bidirectional association between daily physical activity and sleep in older adults^23,26,41^.

In the day-level analysis (level 1), we observed that 10% increase in daytime sedentary behavior was associated with 0.82% increase in REM sleep that night. Furthermore, the participant-level (level 2) analysis revealed that REM latency, stage N3 sleep, and delta power were lower by 8.08 min, 0.46%, and 3344.1 μV^2^, respectively (Fig. 2). However, when low-intensity physical activity during the daytime was higher by 10%, REM sleep duration was lower by 4.80%, and REM latency and the duration of stage N3 sleep were higher by 7.53 min and 0.56% at the day level (level 1), respectively (Fig. 3). In addition, when moderate-to-vigorous-intensity physical activity during daytime was higher by 10%, REM latency was lower by 12.15 min at the day level (level 1). Furthermore, for every 10-point increase in subjective sleep length, daytime moderate-to-vigorous-intensity physical activity during the next day was higher by 0.77% at the day level (level 1) (Fig. 4).

The participant-level (level 2) analysis indicated that higher levels of daytime sedentary behavior are negatively associated with deep sleep parameters (e.g., N3, delta power), while higher low-intensity physical activity during the daytime is positively associated with these parameters. These analyses also revealed that higher delta power during the prior day is negatively associated with daytime sedentary behavior the next day. These findings are in accordance with those of our previous study, which demonstrated that replacing 30 min of sedentary behavior per day with an equal period of low-intensity physical activity may favorably influence objective and subjective sleep parameters^7^. Furthermore, the positive associations between low-intensity physical activity and deep sleep parameters (N3 and delta power) in this study were based on complete polysomnography data.

Although we examined different sleep parameters using actigraphy, a previous study suggests that greater sleep efficiency and lower sleep fragmentation are related to greater levels of moderate-to-vigorous-intensity physical activity the following day^23^. The present study did not show significant differences in the relationship between moderate-to-vigorous-intensity physical activity that resulted in sleep efficiency (B = 0.02, *P* = 0.914 in Model 2; B = − 0.14, *P* = 0.084 in Model 1; Supplementary Table S5) and wake after sleep onset (B = − 0.89, *P* = 0.239 in Model 2; B = − 0.36, *P* = 0.314 in Model 1; Supplementary Table S5). In contrast to the objective sleep parameters, the subjectively long sleep length resulted in increased next-day moderate-vigorous-intensity physical activity (Fig. 4). Unfortunately, the underlying mechanism remains unclear, but depressive symptoms may mediate the relationship between good subjective sleep length and higher moderate-to-vigorous-intensity physical activity (e.g., exercise)^42^. Future studies should consider interventional designs to examine the effect of increasing subjective sleep length on moderate-to-vigorous-intensity physical activity.

Low-intensity aerobic exercise induces an increase in delta power during the first hour of sleep time when compared with sedentary behavior^11^. Although our previous study demonstrated that the same low-intensity level of housework could not induce delta oscillations during sleep among older adults, the current study suggests that higher levels of low-intensity physical activity during the daytime are associated with increases in delta power. Furthermore, combining low-intensity physical activity with social interaction has been found to increase the prevalence of slow-wave sleep^43^. Some researchers have postulated that engaging in low-intensity physical activity in the context of social relationships increases blood flow to the brain, improves neuronal function, and facilitates deep sleep^6,43^. These results suggest that, when compared with moderate-to-vigorous-intensity physical activity, low-intensity physical activity induces positive effects on deep sleep among older adults. Moreover, previous studies have argued for the effects of exercise duration (e.g., acute and chronic effects) on sleep quality^27,44^. This study suggests that low-intensity physical activity, including exercise, has both acute and chronic positive effects on sleep quality in older adults at both the participant and day levels.

Additionally, when compared with objective sleep parameters, sedentary behavior and the intensity of physical activity are associated with fewer predictive factors related to subjective sleep parameters (e.g., OSA-MA). As mentioned above, some studies have argued that there is a mismatch between subjective and objective sleep assessments, proposing that observation periods should last for at least 14 days to resolve these inconsistencies^17,26^.

Although the precise mechanisms underlying the relationship between sedentary behavior and sleep quality remain unclear, previous studies have demonstrated a negative correlation between sedentary behavior and total sleep time/sleep efficiency^44,45^. In addition, the results regarding the appropriate intensity of physical activity remain inconclusive. An epidemiological study has demonstrated that lower-intensity physical activity is better than moderate-to-vigorous-intensity physical activity for slowing or halting the degradation of sleep quality^8^. Our previous epidemiological study also indicated that physically active older adults with robust social networks have a lower prevalence of sleep disorders^6^. Low-intensity physical activity may be positively associated with social relationships since it often involves routine daily social activities, such as caring for grandchildren and walking while having a conversation with others^46^.

However, the present results show a contrary hypothesis that sedentary behavior is positively associated with REM sleep, while the opposite was observed for low-intensity physical activity. In terms of REM latency, sedentary behavior shifted REM sleep forward, while low- and moderate-to-vigorous-intensity physical activity delayed the onset of REM. One meta-analysis concluded that acute physical activity delays REM latency and decreases REM sleep duration, and these changes are the mechanism through which antidepressant treatments work^47^.

An individual’s discretionary time is limited to 24 h per day, meaning that each activity (e.g., sedentary behavior, low-intensity physical activity, moderate-to-vigorous-intensity physical activity, and sleep period) is mutually exclusive. Sedentary behavior and physical activity may exhibit an interdependent relationship with REM latency and REM sleep duration. Indeed, in the present study, REM sleep accounted for 23.3% of the total sleep time, which is similar to the proportion observed among younger people^48^.

The above-mentioned REM sleep among older adults was divided into the following categories: (1) Q1: < 14.8%; (2) Q2: 14.8–19.4%; (3) Q3: 19.5–23.6%; and (4) Q4: > 23.6%^19^. Notably, REM sleep is fundamentally different from non-REM sleep given that it is associated with a higher metabolic rate, higher heart rate, activation of the sympathetic nervous system, and patterns of global brain activity similar to those observed during wakefulness^49,50^. Indeed, the duration of REM sleep exhibited a significant negative correlation with the self-reported frequency of dreaming (*r* = − 0.211, *P* = 0.044) in this study. Furthermore, patients with depression exhibit early appearance of REM latency and a higher rate of REM sleep^51^, and REM sleep of ≥ 41% of the total sleep time indicates bad sleep quality among older adults^37^. Since human sleep time is also limited, the present study suggests that low-intensity physical activity could shift REM sleep to a deep N3 sleep stage, and both low- and moderate-to-vigorous-intensity physical activity could delay REM onset. There was also no significant association between depressive symptoms and REM latency or REM sleep in the current study (Supplementary Tables S1–S21). However, as this study included only healthy older adults, future studies should focus on patients with depression and/or insomnia.

The present study overcomes the limitations of previous studies using polysomnography; however, it has other limitations. First, this micro-longitudinal observational study was conducted during the coronavirus disease 2019 (COVID-19) pandemic. It is possible that restrictions on or avoidance of physical activity due to the COVID-19 pandemic affected physical activity patterns, although the levels of physical activity observed appear similar to those reported among US and Japanese older adults prior to the COVID-19 pandemic^52,53^. Second, a previous study reported that there are sex-based differences in sleep quality^54^. Additionally, we could not investigate the participants’ educational levels. Although our analyses were adjusted for sex and other potential confounders, future studies should focus on individual differences, including sex and education level. Moreover, a similar study has considered the weekday/weekend sleep patterns among students^22^. However, no significant differences were noted between the midpoint of sleep time on weekday and that on weekend (e.g., social jet lag) in the older adults in the present study (Supplementary Fig. S1). Third, the sample size was small, despite the use of objective data obtained using accelerometers and polysomnography. Fourth, as mentioned above, every physical activity has strong relevance to each other. Our previous study showed the influence of replacing sedentary behavior with engaging or other intensities of physical activities (e.g., low- and moderate-to-vigorous intensity physical activity) on sleep quality^7^. However, autocorrelation could not be considered in our study because there is multi-collinearity when multi-level analysis inserting the level 1 and level 2 variables of activities was undertaken. In addition, this study examined bidirectional associations in two separate models. Although our models tested two temporal directions (sedentary/physical activities to sleep that night, and sleep to next-day sedentary/physical activities), these associations are likely to be correlational. We do not yet know whether each variable (physical activity or sleep) is associated with change in the other. Future studies may need to use more rigorous models that control for the prior day’s behavior (physical activity and/or sleep). Finally, although we excluded participants who had taken sleeping medications, some patients in our study may have had obstructive sleep apnea despite a lack of subjective symptoms. To verify our results, future investigations with larger sample sizes in older adults are necessary.

In conclusion, our results suggest that there are bidirectionally negative associations between sedentary behavior and delta power at the participant level. Contrarily, more low-intensity physical activity during the daytime is associated with greater deep sleep. Moreover, longer subjective sleep length is associated with higher moderate-to-vigorous-intensity physical activity the next day. However, the number of parameters related to next-day physical activity predicted by sleep in the present study was fewer than in previous studies. Meanwhile, the results of the present study suggest that regardless of intensity, engaging in physical activity will increase REM latency, while low-intensity physical activity will increase deep sleep and decrease REM sleep in older adults.

## Methods

### Participants

This micro-longitudinal observational study was conducted from February to June 2021. A total of 112 older adults were recruited from January to May 2021 through advertisements and snowball sampling in Ibaraki Prefecture, Japan. The inclusion criteria were age over 65 years and having no restrictions on exercise as imposed by a physician. The participants were required to wear an accelerometer during the daytime for 7 consecutive days and to undergo polysomnography during the sleep period. In addition, to assess self-reported sleep quality, the participants were instructed to complete a detailed questionnaire after waking up for 7 consecutive days. For the assessment of usual daily physical activity and sleep habits, the participants were required to spend the day how they normally would have, and we did not control the total sleep time, bedtime, and awake time.

We excluded individuals who were taking sleeping medication (n = 7) and those who provided less than 3 days of paired accelerometer and polysomnography data (n = 13). Finally, daily physical activity and polysomnography data were analyzed for 92 participants (Table 1). Except for that in alcohol consumption, no significant differences were noted in the characteristics between the included and excluded participants (Supplementary Table S22). All participants provided written informed consent and were compensated with 4000 Japanese yen. This study was performed in accordance with the principles of the Declaration of Helsinki and the Ethics Committee of the University of Tsukuba approved this study (reference no. R02-211).

### Measurements

#### Polysomnography assessment and self-reported sleep parameters

Objective daily sleep parameters were recorded using a wearable polysomnography device (Insomnograf K1; S’UIMIN Inc., Tokyo, Japan), which is light (162 g) and easy to attach and remove due to the soft sticking electrodes, making it easy to use in older adults. A pilot study found an 86.9% concordance rate and 0.80 kappa coefficient with a typical polysomnography device. Participants were instructed to attach electrodes to their head after showering and to push the record button immediately before going to bed and after waking up. The time to bed and last awakening in the morning were determined using these data. The participants pushed the power button or inserted the cable for charge, and the recording ended. We cross-checked the bedtime and wake-up time recorded by polysomnography and the self-reported questionnaires. The two instruments were used together to ensure that data were collected even in cases when participants forgot to push the button immediately after waking up. Although, some minor differences were noted in bedtime and waketime, there was a high correlation between the polysomnography and self-reported questionnaires on bedtime (*r* = 0.935, *P* < 0.001), waketime (*r* = 0.933, *P* < 0.001), and midpoint of sleep time (*r* = 0.967, *P* < 0.001).

This device’s recording system consisted of five electroencephalogram derivations (Fp1–M2, Fp2–M1, Fp1–average M, Fp2–average M, and Fp1–Fp2). The records were scored every 30 s to classify sleep stages as wakefulness (stage W); non-REM (stage N); N1, N2, and N3; and REM. Measurements during sleep onset latency were classified as stage W. Moreover, REM latency was measured until the first REM stage that appeared after sleep onset. Waking after sleep onset was defined according to standard criteria^55^. The Fp1 to average M recording was analyzed using discrete fast Fourier transform techniques, as previously described^27^. The total delta [0.75–4.00 Hz] power was calculated during non-REM sleep; the power content of the delta band was reported as a 5-s epoch of the sleep phase^27^ in μV^2^. We calculated the sum of delta power during the night because we could not control participants’ total sleep time in their home environment.

The Oguri–Shirakawa–Azumi sleep inventory, Middle-Aged and Aged version (OSA-MA) is a questionnaire designed to assess self-reported sleep quality the next morning^56^. The participants used a pen to write in a printed form due to the difficulty encountered by older adults in using and/or the absence of a smartphone. This questionnaire consists of bedtime/waketime and 16 components, each of which is graded into four levels (1–4 score) and divided into five categories: sleepiness on rising, initiation and maintenance of sleep, frequent dreaming, feeling refreshed, and sleep length. The sum of the scores for each category is used to determine the global score. Higher scores indicate better sleep quality. The reliability and reproducibility of the OSA-MA have been confirmed in a previous study^56^.

#### Daily sedentary behavior and intensity of physical activity

Sedentary behavior and physical activity were measured using a triaxial accelerometer (GT3X-BT; ActiGraph, Pensacola, FL). Participants wore an accelerometer on their waist during all waking hours, except when either changing clothes or bathing. Accelerometer data were collected at a sampling rate of 30 Hz, aggregated over 60-s intervals, and analyzed using ActiLife (version 6.13.4). The time spent not wearing the device (non-wearing time) was recorded at intervals of at least 60 consecutive minutes of zero counts. Wearing time was determined by subtracting the total non-wearing time from 24 h. Based on a previous study^32^, the count per minute (cpm) data were divided into three levels: (1) sedentary behavior (less than 50 cpm), (2) low-intensity physical activity (51–1040 cpm), and (3) moderate-to-vigorous-intensity physical activity (more than 1041 cpm)^53,57^. Days with over 10 h of wearing time were considered valid^7^.

#### Potential confounders

Based on previous studies^58^, we included age (continuous variable), sex (male or female), body mass index (BMI) (continuous variable), medical history (hypertension, hyperlipidemia; yes or no), caffeine consumption (daily or 1–6 times per week/less than 1–3 times per month), alcohol consumption (daily or 1–6 times per week/less than 1–3 times per month), tobacco smoking status (current or past/never), and depressive symptoms as confounders. Interviews and BMI assessments were conducted during the study visit by trained students. Depressive symptoms were assessed using the Japanese version of the 15-item Geriatric Depression Scale^59^ (continuous variable).

### Temporal alignment of sleep and physical activity measures

To examine the bidirectional associations between sedentary/physical activities and sleep, we developed two datasets. First, when we tested temporal associations of sedentary/physical activities predicting that night’s sleep, the data set consisted of data on physical activity and sleep parameters of that day (587 daily observations were clustered from 92 older adults). Second, when we confirmed the temporal associations of each night’s sleep predicting the next day sedentary/physical activities, the data set consisted of sleep parameters coupled with the next day sedentary/physical activity data (489 daily observations were clustered within 92 older adults).

### Statistical analysis

Multilevel models with lagged effects were analyzed using R software (R Foundation for Statistical Computing, Vienna, Austria). First, the precondition of the multilevel model analysis was a hierarchical model of all the variables. Thus, the null model was used to confirm the intra-class correlation (ICC) for all variables. The ICC was 0.36–0.83 through sedentary/physical activities and sleep variables (Table 2). We developed a two-level model to examine the associations between daily physical activity and nighttime sleep measures according to between-participant (participant level: level 2) and within-participant (day level: level 1) levels. The independent variables were centered at the sample mean for the between-participant variables, and the within-participant variables were centered at the person mean. Additionally, to investigate the bidirectional associations between daily physical activity and nighttime sleep measures, we used the two datasets mentioned above. Potential confounders were included in all models (Supplementary Tables S1–S21). Statistical significance was set at *P* < 0.05 (two-tailed test).

**Table 2:** Null model without independent variables on objective sleep parameters.

Variables	*β* Coefficients			
	Intercept	Within participant	Between participant	ICC
**Polysomnography**				
Total sleep time, min	373.8	2361.5	1582.2	0.40
Sleep latency, min	19.7	162.4	92.8	0.36
Sleep efficiency, %	81.4	48.6	30.5	0.39
WASO, min	68.1	889.4	781.4	0.47
REM latency, min	96.8	1371.1	969.8	0.41
N1, %	10.5	7.9	16.1	0.67
N2, %	58.3	25.7	44.6	0.63
N3, %	7.9	12.1	51.6	0.81
REM, %	23.3	20.5	13.3	0.39
Sum of delta power, μV^2^/night	115,386	678,567,863	2,891,874,440	0.83
**OSA-MA**				
Sleepiness on rising, pts	51.8	31.1	51.4	0.62
Initiation and maintenance of sleep, pts	46.8	65.4	44.4	0.40
Frequent dreaming, pts	52.5	44.3	31.7	0.42
Refreshing, pts	53.2	45.8	42.6	0.48
Sleep length, pts	48.3	48.8	38.1	0.44
Total score, pts	252.6	527.6	569.8	0.52
**Physical activity**				
SB, %	58.2	54.7	87.2	0.61
LPA, %	34.7	33.7	46.7	0.58
MVPA, %	7.1	16.1	20.2	0.56

*ICC* intraclass correlation coefficients, *WASO* wake after sleep onset, *REM* rapid eye movement, *N* non-REM sleep stage, *OSA-MA* Oguri–Shirakawa–Azumi sleep inventory, Middle-Aged and Aged version, *SB* sedentary behavior, *LPA* low-intensity physical activity, *MVPA* moderate-vigorous-intensity physical activity. 587 polysomnography data were clustered within 92 older adults.

## Supplementary Information

Supplementary Information.

## Data Availability

The datasets used and/or analyzed during the current study are available from the corresponding author (seol.jaehoon.ge@u.tsukuba.ac.jp) on reasonable request.
