# Supplementary material for: Bidirectional associations between physical activity and sleep in older adults: a multilevel analysis using polysomnography
Source: Sci Rep. 2022 Sep 13;12:15399. doi: 10.1038/s41598-022-19841-x (PMC9470065; doi:10.1038/s41598-022-19841-x)
Supplement: Supplementary file 1 — Supplementary Information. [file 41598_2022_19841_MOESM1_ESM.docx]

**Supplementary Information**

Supplementary Information for **Bidirectional associations between physical activity and sleep in older adults: a multilevel analysis using polysomnography**

Jaehoon Seol, Jaehee Lee, Insung Park, Kumpei Tokuyama, Shoji Fukusumi, Toshio Kokubo, Masashi Yanagisawa, and Tomohiro Okura

Correspondence should be addressed to Jaehoon Seol, PhD.

Email: seol.jaehoon.ge@u.tsukuba.ac.jp

**This PDF file includes:**

Table S1 to S22

Fig. S1

Supplementary Table S1. Results from multilevel models of daytime sedentary behavior predicting night polysomnography parameters

|  | *β* Coefficients (SE) | | | | | | | | | |
| --- | --- | --- | --- | --- | --- | --- | --- | --- | --- | --- |
|  | Total sleep time, min | Sleep latency, min | Sleep efficiency, % | Wake after sleep onset, min | REM latency, min | N1, % | N2, % | N3, % | R, % | Sum of delta power, μV^2^ /night |
| Intercept | **378.73**  **(84.99)** | 28.87  (21.79) | **87.32**  **(11.50)** | 49.99  (57.95) | 8.65  (66.86) | **17.34**  **(8.15)** | 30.37  (13.27) | 4.95  (13.60) | **46.80**  **(7.86)** | 54012  (103981) |
| Age | 0.1  (1.0) | -0.04  (0.25) | -0.18  (0.13) | 0.73  (0.66) | 1.40  (0.77) | -0.04  (0.09) | 0.41  (0.15) | -0.07  (0.16) | **-0.29**  **(0.09)** | -406  (1192) |
| Sex  (Ref: Male) | -11.84 (13.30) | -1.74  (3.41) | **3.63**  **(1.80)** | **-19.80**  **(9.08)** | -6.14  (10.47) | -1.41  (1.28) | -0.74  (2.08) | 3.65  (2.13) | -1.50  (1.23) | **46685**  **(16310)** |
| BMI | 1.11  (5.30) | 1.88  (1.36) | -0.79  (0.72) | 2.16  (3.61) | 5.12  (4.17) | 0.11  (0.51) | 0.37  (0.83) | -0.07  (0.85) | -0.39  (0.49) | 125  (6473) |
| Smoking history  (Ref: Past/never) | -73.12  (51.46) | 12.65  (13.25) | -5.02  (7.04) | -4.00  (34.57) | 18.30  (40.26) | 3.92  (4.69) | 4.56  (7.68) | -12.88  (7.71) | 4.30  (4.76) | -113381  (58921) |
| Alcohol consumption  (Ref: Less than 1-6 time per week) | 5.57  (10.26) | -2.63  (2.63) | 1.43  (1.39) | -5.27  (7.01) | -11.06  (8.08) | 1.04  (0.99) | 0.46  (1.61) | -1.61  (1.65) | 0.12  (0.95) | -5567  (12584) |
| Caffeine consumption  (Ref: Less than 1-6 time per week) | 4.11  (17.44) | -3.24  (4.48) | 0.24  (2.36) | 2.63  (11.89) | -5.15  (13.73) | -1.88  (1.67) | -1.53  (2.72) | 3.10  (2.78) | 0.48  (1.62) | 16007  (21251) |
| Hypertension  (Ref: No) | -14.34  (10.50) | 3.30  (2.69) | -1.86  (1.42) | 4.03  (7.17) | -4.90  (8.27) | 1.21  (1.01) | 1.90  (1.64) | **-4.25**  **(1.69)** | 1.14  (0.97) | -11420  (12907) |
| Hyperlipidemia  (Ref: No) | **32.59**  **(12.88)** | -6.30  (3.31) | **4.84**  **(1.74)** | -15.03  (8.80) | 10.77  (10.15) | -1.33  (1.24) | -2.15  (2.02) | 3.55  (2.07) | -0.15  (1.19) | 6978  (15832) |
| Depressive symptoms | -0.87  (9.13) | -0.89  (2.34) | 0.94  (1.24) | -4.22  (6.23) | 7.77  (7.19) | -0.44  (0.88) | -0.01  (1.43) | 0.68  (1.47) | -0.27  (0.85) | -3740  (11199) |
| SB  (Between-person) | 0.11  (0.61) | 0.06  (0.16) | -0.03  (0.08) | 0.28  (0.40) | -0.71  (0.47) | -0.01  (0.05) | 0.06  (0.09) | **-0.19**  **(0.09)** | **0.14**  **(0.06)** | **-1473**  **(659)** |
| SB  (Within-person) | -0.12  (0.31) | -0.03  (0.08) | 0.02  (0.04) | 0.09  (0.19) | **-0.81**  **(0.23)** | 0.01  (0.02) | -0.05  (0.03) | **-0.05**  **(0.02)** | **0.08**  **(0.03)** | **-334**  **(162)** |

Note: Bold numbers indicate *P* < .05; SE, standard error; BMI, body mass index; SB, sedentary behavior.

Supplementary Table S2. Results from multilevel models of daytime sedentary behavior predicting subjective sleep quality

|  | *β* Coefficients (SE) | | | | | |
| --- | --- | --- | --- | --- | --- | --- |
|  | Sleepiness on rising | Initiation and maintenance of sleep | Frequent dreaming | Refreshing | Sleep length | OSA-MA total score |
| Intercept | **67.61**  **(14.46)** | **50.64**  **(14.44)** | **49.96**  **(12.09)** | **56.57**  **(14.10)** | **48.16**  **(13.56)** | **272.95**  **(51.32)** |
| Age | -0.22  (0.17) | -0.08  (0.17) | 0.03  (0.14) | 0.01  (0.16) | -0.06  (0.16) | -0.31  (0.59) |
| Sex  (Ref: Male) | -2.90  (2.26) | -1.02  (2.26) | 2.28  (1.89) | -1.77  (2.20) | 1.44  (2.12) | -1.97  (8.02) |
| BMI | -0.23  (0.90) | 0.58  (0.90) | 1.30  (0.75) | -0.38  (0.88) | 0.39  (0.84) | 1.65  (3.19) |
| Smoking history  (Ref: Past/never) | -4.71  (8.68) | -3.63  (9.35) | 8.59  (7.78) | 6.16  (8.80) | 2.00  (8.56) | 8.39  (31.54) |
| Alcohol consumption  (Ref: Less than 1-6 time per week) | -1.03  (1.75) | -2.19  (1.75) | -0.79  (1.47) | -1.39  (1.71) | -1.05  (1.64) | -6.39  (6.22) |
| Caffeine consumption  (Ref: Less than 1-6 time per week) | **7.32**  **(2.93)** | 5.23  (2.93) | **-5.00**  **(2.45)** | 0.70  (2.86) | 2.24  (2.75) | 10.49  (10.41) |
| Hypertension  (Ref: No) | -1.62  (1.79) | 0.30  (1.79) | 1.13  (1.50) | -1.88  (1.75) | 1.44  (1.68) | -0.62  (6.35) |
| Hyperlipidemia  (Ref: No) | 1.76  (2.19) | -3.19  (2.19) | -2.39  (1.83) | 1.47  (2.14) | -0.13  (2.05) | -2.47  (7.78) |
| Depressive symptoms | -1.30  (1.58) | -0.12  (1.58) | -0.18  (1.32) | -1.91  (1.54) | **-3.09**  **(1.48)** | -6.59  (5.60) |
| SB  (Between-person) | 0.04  (0.10) | 0.12  (0.10) | 0.01  (0.08) | 0.07  (0.10) | -0.01  (0.09) | 0.23  (0.34) |
| SB  (Within-person) | -0.00  (0.03) | -0.02  (0.05) | -0.06  (0.04) | -0.00  (0.04) | -0.05  (0.04) | -0.14  (0.14) |

Note: Bold numbers indicate *P* < .05; SE, standard error; BMI, body mass index; SB, sedentary behavior; OSA-MA, Oguri-Shirakawa-Azumi sleep inventory, Middle-Aged and Aged version.

|  | *β* Coefficients (SE) | | | | | | | | | |
| --- | --- | --- | --- | --- | --- | --- | --- | --- | --- | --- |
|  | Total sleep time, min | Sleep latency, min | Sleep efficiency, % | Wake after sleep onset, min | REM latency, min | N1, % | N2, % | N3, % | R, % | Sum of delta power, μV^2^ /night |
| Intercept | **379.87**  **(86.58)** | 26.23  (22.27) | **86.16**  **(11.76)** | 47.99  (59.08) | -4.45  (67.94) | **17.35**  **(8.30)** | **31.31**  **(13.56)** | 2.77  (13.86) | **47.92**  **(7.99)** | 32246  (105578) |
| Age | 0.13  (0.98) | -0.02  (0.25) | -0.18  (0.13) | 0.75  (0.67) | 1.47  (0.77) | -0.04  (0.09) | **0.40**  **(0.15)** | -0.06  (0.16) | **-0.30**  **(0.09)** | -298  (1194) |
| Sex  (Ref: Male) | -12.09  (13.75) | -1.20  (3.54) | 3.46  (1.87) | **-19.44**  **(9.38)** | -3.26  (10.79) | -1.41  (1.32) | -0.94  (2.16) | 4.13  (2.21) | -1.75  (1.27) | **51428**  **(16799)** |
| BMI | 1.00  (5.29) | 1.80  (1.36) | -0.76  (0.72) | 2.03  (3.61) | 4.87  (4.15) | 0.12  (0.51) | 0.33  (0.83) | -0.03  (0.85) | -0.40  (0.49) | 1489  (6444) |
| Smoking history  (Ref: Past/never) | -74.79  (52.01) | 9.56  (13.41) | -3.94  (7.13) | -8.04  (34.94) | 5.98  (40.60) | 4.20  (4.74) | 4.17  (7.79) | -13.21  (7.80) | 4.69  (4.80) | **-119458**  **(59425)** |
| Alcohol consumption  (Ref: Less than 1-6 time per week) | 5.62  (10.29) | -2.49  (2.65) | 1.38  (1.40) | -5.12  (7.02) | -10.42  (8.07) | 1.03  (0.99) | 0.46  (1.61) | -1.56  (1.65) | 0.09  (0.95) | -4961  (12570) |
| Caffeine consumption  (Ref: Less than 1-6 time per week) | 3.72  (17.78) | -2.78  (4.57) | 0.10  (2.41) | 2.86  (12.12) | -2.79  (13.95) | -1.87  (1.70) | -1.79  (2.78) | 3.60  (2.83) | 0.24  (1.64) | 20866  (21572) |
| Hypertension  (Ref: No) | -14.53  (10.63) | 2.75  (2.74) | -1.65  (1.44) | 3.43  (7.26) | -7.04  (8.35) | 1.25  (1.02) | 1.88  (1.67) | **-4.34**  **(1.71**) | 1.21  (0.98) | -12842  (13020) |
| Hyperlipidemia  (Ref: No) | **32.40**  **(13.33)** | -5.73  (3.43) | **4.65**  **(1.81)** | -14.61  (9.11) | 13.73  (10.47) | -1.33  (1.28) | -2.33  (2.10) | 4.01  (2.15) | -0.40  (1.23) | 11622  (16347) |
| Depressive symptoms | -0.68  (9.15) | -0.57  (2.35) | 0.82  (1.24) | -3.85  (6.25) | 8.94  (7.18) | -0.47  (0.89) | 0.04  (1.44) | 0.69  (1.47) | -0.29  (0.85) | -3339  (11185) |
| LPA  (Between-person) | -0.09  (0.86) | -0.02  (0.22) | 0.04  (0.12) | -0.07  (0.58) | 1.21  (0.67) | <-0.01  (0.08) | -0.05  (0.13) | **0.26**  **(0.13)** | **-0.19**  **(0.08)** | **2153**  **(959)** |
| LPA  (Within-person) | 0.14  (0.38) | -0.05  (0.10) | 0.03  (0.06) | 0.01  (0.23) | **0.75**  **(0.29)** | -0.02  (0.02) | 0.07  (0.34) | **0.06**  **(0.03)** | **-0.10**  **(0.04)** | 353  (203) |

Supplementary Table S3. Results from multilevel models of daytime low-intensity physical activity predicting night polysomnography parameter

Note: Bold numbers indicate *P* < .05; SE, standard error; BMI, body mass index; LPA, low-intensity physical activity.

|  | *β* Coefficients (SE) | | | | | |
| --- | --- | --- | --- | --- | --- | --- |
|  | Sleepiness on rising | Initiation and maintenance of sleep | Frequent dreaming | Refreshing | Sleep length | OSA-MA total score |
| Intercept | **69.30**  **(14.67)** | **53.78**  **(14.60)** | **52.21**  **(12.22)** | **56.78**  **(14.38)** | **50.94**  **(13.69)** | **283.03**  **(51.86)** |
| Age | -0.23  (0.17) | -0.09  (0.17) | 0.02  (0.14) | 0.01  (0.16) | -0.07  (0.16) | -0.36  (0.59) |
| Sex  (Ref: Male) | -3.27  (2.33) | -1.71  (2.31) | 1.79  (1.94) | -1.82  (2.28) | 0.84  (2.17) | -4.17  (8.22) |
| BMI | -0.23  (0.89) | 0.55  (0.89) | 1.30  (0.75) | -0.41  (0.88) | 0.42  (0.84) | 1.64  (3.17) |
| Smoking history  (Ref: Past/never) | -3.58  (8.76) | -2.45  (9.39) | 9.88  (7.82) | 5.78  (8.90) | 4.03  (8.60) | 13.68  (31.71) |
| Alcohol consumption  (Ref: Less than 1-6 time per week) | -1.08  (1.75) | -2.27  (1.74) | -0.86  (1.46) | -1.37  (1.72) | -1.15  (1.63) | -6.68  (6.19) |
| Caffeine consumption  (Ref: Less than 1-6 time per week) | **6.95**  **(2.98)** | 4.51  (2.96) | **-5.49**  **(2.48)** | 0.61  (2.92) | 1.67  (2.78) | 8.24  (10.53) |
| Hypertension  (Ref: No) | -1.42  (1.81) | 0.58  (1.80) | 1.39  (1.51) | -1.97  (1.77) | 1.83  (1.69) | 0.43  (6.39) |
| Hyperlipidemia  (Ref: No) | 1.39  (2.26) | -3.88  (2.25) | -2.88  (1.89) | 1.42  (2.22) | -0.75  (2.11) | -4.68  (8.00) |
| Depressive symptoms | -1.39  (1.58) | -0.23  (1.57) | -0.30  (1.32) | -1.85  (1.55) | **-3.30**  **(1.47)** | -7.06  (5.58) |
| LPA  (Between-person) | -0.14  (0.14) | -0.20  (0.14) | -0.09  (0.12) | -0.13  (0.14) | -0.08  (0.13) | -0.65  (0.49) |
| LPA  (Within-person) | -0.03  (0.04) | 0.04  (0.06) | 0.06  (0.05) | -0.06  (0.05) | 0.06  (0.05) | 0.07  (0.18) |

Supplementary Table S4. Results from multilevel models of daytime low-intensity physical activity predicting subjective sleep quality

Note: Bold numbers indicate *P* < .05; SE, standard error; BMI, body mass index; LPA, low-intensity physical activity; OSA-MA, Oguri-Shirakawa-Azumi sleep inventory, Middle-Aged and Aged version.

|  | *β* Coefficients (SE) | | | | | | | | | |
| --- | --- | --- | --- | --- | --- | --- | --- | --- | --- | --- |
|  | Total sleep time, min | Sleep latency, min | Sleep efficiency, % | Wake after sleep onset, min | REM latency, min | N1, % | N2, % | N3, % | R, % | Sum of delta power, μV^2^ /night |
| Intercept | **371.76**  **(84.30)** | 24.98  (21.43) | **88.98**  **(11.33)** | 43.80  (57.44) | 1.06  (65.62) | **18.02**  **(8.09)** | **27.46**  **(13.21)** | 8.67  (13.65) | **45.36**  **(7.83)** | 82771  (104636) |
| Age | 0.18  (0.97) | -0.02  (0.25) | -0.19  (0.13) | 0.76  (0.66) | 1.42  (0.76) | -0.05  (0.09) | **0.42**  **(0.15)** | -0.09  (0.16) | **-0.28**  **(0.09)** | -584  (1204) |
| Sex  (Ref: Male) | -10.37  (13.16) | -0.94  (3.35) | 3.26  (1.76) | **-18.42**  **(8.97)** | -4.43  (10.24) | -1.56  (1.26) | -0.11  (2.06) | 2.88  (2.13) | -1.22  (1.22) | **40659**  **(16340)** |
| BMI | 1.14  (5.30) | 1.99  (1.35) | -0.81  (0.71) | 2.24  (3.61) | 5.65  (4.12) | 0.10  (0.51) | 0.37  (0.83) | -0.03  (0.86) | -0.42  (0.49) | 1727  (6559) |
| Smoking history  (Ref: Past/never) | -76.39  (49.65) | 12.69  (12.68) | -4.83  (6.75) | -5.68  (33.26) | 25.17  (38.47) | 4.17  (4.50) | 2.90  (7.41) | -9.90  (7.48) | 2.81  (4.61) | -87406  (57292) |
| Alcohol consumption  (Ref: Less than 1-6 time per week) | 5.79  (10.24) | -2.58  (2.60) | 1.42  (1.37) | -5.11  (6.98) | -11.17  (7.97) | 1.02  (0.98) | 0.57  (1.61) | -1.77  (1.66) | 0.19  (0.95) | -6883  (12723) |
| Caffeine consumption  (Ref: Less than 1-6 time per week) | 5.60  (17.43) | -2.27  (4.43) | -0.14  (2.34) | 4.00  (11.86) | -2.81  (13.56) | -2.03  (1.67) | -0.93  (2.72) | 2.39  (2.81) | 0.73  (1.62) | 10654  (21507) |
| Hypertension  (Ref: No) | -15.07  (10.23) | 3.17  (2.60) | -1.79  (1.37) | 3.66  (6.98) | -4.54  (7.97) | 1.27  (0.98) | 1.56  (1.61) | **-3.72**  **(1.66)** | 0.92  (0.95) | -6911  (12732) |
| Hyperlipidemia  (Ref: No) | **34.02**  **(12.69)** | -5.57  (3.23) | **4.51**  **(1.70)** | -13.71  (8.65) | 12.18  (9.88) | -1.48  (1.22) | -1.53  (1.99) | 2.75  (2.06) | 0.14  (1.18) | 729  (15793) |
| Depressive symptoms | -0.57  (9.04) | -0.94  (2.30) | 0.93  (1.22) | -4.14  (6.16) | 7.18  (7.04) | -0.46  (0.87) | 0.15  (1.42) | 0.43  (1.47) | -0.15  (0.84) | -6051  (11240) |
| MVPA  (Between-person) | -0.32  (1.14) | -0.15  (0.29) | 0.02  (0.15) | -0.89  (0.76) | 0.08  (0.88) | 0.06  (0.10) | -0.15  (0.17) | 0.21  (0.17) | -0.11  (0.11) | 1502  (1264) |
| MVPA  (Within-person) | 0.13  (0.13) | 0.24  (0.15) | -0.14  (0.08) | -0.36  (0.36) | **1.21**  **(0.45)** | 0.01  (0.03) | 0.02  (0.06) | 0.04  (0.04) | -0.06  (0.05) | 399  (314) |

Supplementary Table S5. Results from multilevel models of daytime moderate-vigorous-intensity physical activity predicting night polysomnography parameter

Note: Bold numbers indicate *P* < .05; SE, standard error; BMI, body mass index; MVPA, moderate-vigorous-intensity physical activity.

Supplementary Table S6. Results from multilevel models of daytime moderate-vigorous-intensity physical activity predicting subjective sleep quality

|  | *β* Coefficients (SE) | | | | | |
| --- | --- | --- | --- | --- | --- | --- |
|  | Sleepiness on rising | Initiation and maintenance of sleep | Frequent dreaming | Refreshing | Sleep length | OSA-MA total score |
| Intercept | **66.66**  **(14.39)** | **47.31**  **(14.53)** | **48.58**  **(12.07)** | **54.38**  **(14.00)** | **47.85**  **(13.45)** | **264.82**  **(51.39)** |
| Age | -0.21  (0.17) | -0.06  (0.17) | 0.04  (0.14) | 0.02  (0.16) | -0.05  (0.16) | -0.26  (0.59) |
| Sex  (Ref: Male) | -2.70  (2.24) | -0.32  (2.26) | 2.56  (1.88) | -1.29  (2.18) | 1.48  (2.09) | -0.24  (7.99) |
| BMI | -0.28  (0.90) | 0.51  (0.91) | 1.25  (0.76) | -0.38  (0.88) | 0.32  (0.84) | 1.42  (3.21) |
| Smoking history  (Ref: Past/never) | -6.15  (8.39) | -6.90  (9.15) | 6.89  (7.57) | 5.08  (8.50) | 0.38  (8.28) | -0.76  (30.66) |
| Alcohol consumption  (Ref: Less than 1-6 time per week) | -0.96  (1.75) | -2.05  (1.77) | -0.70  (1.47) | -1.31  (1.70) | -0.98  (1.64) | -5.94  (6.26) |
| Caffeine consumption  (Ref: Less than 1-6 time per week) | **7.47**  **(2.93)** | **5.86**  **(2.96)** | -4.78  (2.46) | 1.18  (2.85) | 2.20  (2.74) | 11.93  (10.48) |
| Hypertension  (Ref: No) | -1.87  (1.75) | -0.29  (1.76) | 0.79  (1.46) | -2.11  (1.70) | 1.16  (1.63) | -2.33  (6.23) |
| Hyperlipidemia  (Ref: No) | 1.97  (2.16) | -2.45  (2.18) | -2.09  (1.81) | 1.94  (2.10) | -0.05  (2.02) | -0.64  (7.72) |
| Depressive symptoms | -1.15  (1.56) | 0.21  (1.58) | 0.01  (1.31) | -1.81  (1.52) | **-2.91**  **(1.46)** | -5.64  (7.72) |
| MVPA  (Between-person) | 0.08  (0.18) | -0.11  (0.19) | 0.09  (0.16) | -0.02  (0.18) | 0.14  (0.17) | 0.18  (0.65) |
| MVPA  (Within-person) | 0.07  (0.06) | -0.01  (0.09) | 0.09  (0.07) | 0.13  (0.08) | 0.03  (0.08) | 0.32  (0.25) |

Note: Bold numbers indicate *P* < .05; SE, standard error; BMI, body mass index; MVPA, moderate-vigorous-intensity physical activity; OSA-MA, Oguri-Shirakawa-Azumi sleep inventory, Middle-Aged and Aged version.

Supplementary Table S7. Results from multilevel models of delta power predicting next day sedentary behavior and physical activity

|  | *β* Coefficients (SE) | | |
| --- | --- | --- | --- |
|  | SB | LPA | MVPA |
| Intercept | **81.96**  **(18.29)** | 8.12  (12.24) | 9.81  (9.97) |
| Age | -0.13  (0.21) | 0.13  (0.14) | 0.00  (0.12) |
| Sex  (Ref: Male) | -5.60  (2.97) | **6.44**  **(1.99)** | -0.86  (1.62) |
| BMI | 0.77  (1.15) | -0.41  (0.77) | -0.36  (0.62) |
| Smoking history  (Ref: Past/never) | **34.71**  **(10.66)** | **-26.43**  **(7.31)** | -8.25  (5.85) |
| Alcohol consumption  (Ref: Less than 1-6 time per week) | -1.05  (2.23) | 1.22  (1.49) | -0.18  (1.22) |
| Caffeine consumption  (Ref: Less than 1-6 time per week) | -5.02  (3.78) | **5.69**  **(2.53)** | -0.68  (2.06) |
| Hypertension  (Ref: No) | **5.58**  **(2.22)** | **-4.31**  **(1.48)** | -1.24  (1.21) |
| Hyperlipidemia  (Ref: No) | -4.94  (2.76) | **5.83**  **(1.84)** | -0.91  (1.50) |
| Depressive symptoms | -2.91  (1.96) | 2.25  (1.31) | 0.65  (1.07) |
| Sum of delta power  (Between-person) | **-0.00**  **(0.00)** | 0.00  (0.00) | 0.00  (0.00) |
| Sum of delta power  (Within-person) | -0.00  (0.00) | 0.00  (0.00) | 0.00  (0.00) |

Note: Bold numbers indicate *P* < .05; SE, standard error; BMI, body mass index; SB, sedentary behavior; LPA, low-intensity physical activity; MVPA, moderate-vigorous-intensity physical activity.

Supplementary Table S8. Results from multilevel models of sleep length predicting next day sedentary behavior and physical activity

|  | *β* Coefficients (SE) | | |
| --- | --- | --- | --- |
|  | SB | LPA | MVPA |
| Intercept | **83.38**  **(18.22)** | 7.14  (12.15) | 9.36  (9.69) |
| Age | -0.20  (0.21) | 0.19  (0.14) | 0.01  (0.11) |
| Sex  (Ref: Male) | -4.08  (2.84) | **5.00**  **(1.89)** | -0.93  (1.51) |
| BMI | 0.72  (1.14) | -0.38  (0.76) | -0.33  (0.61) |
| Smoking history  (Ref: Past/never) | **26.68**  **(10.56)** | **-19.75**  **(7.25)** | -6.90  (5.65) |
| Alcohol consumption  (Ref: Less than 1-6 time per week) | -1.02  (2.23) | 1.09  (1.49) | -0.09  (1.18) |
| Caffeine consumption  (Ref: Less than 1-6 time per week) | -3.70  (3.72) | 4.50  (2.48) | -0.80  (1.18) |
| Hypertension  (Ref: No) | **5.42**  **(2.21)** | **-4.49**  **(1.47)** | -0.91  (1.18) |
| Hyperlipidemia  (Ref: No) | -5.05  (2.73) | **5.96**  **(1.82)** | -0.92  (1.45) |
| Depressive symptoms | -2.92  (2.03) | 2.54  (1.36) | 0.36  (1.08) |
| Sleep length  (Between-person) | -0.02  (0.16) | -0.13  (0.11) | 0.16  (0.09) |
| Sleep length  (Within-person) | -0.05  (0.05) | -0.02  (0.04) | **0.08**  **(0.03)** |

Note: Bold numbers indicate *P* < .05; SE, standard error; BMI, body mass index; SB, sedentary behavior; LPA, low-intensity physical activity; MVPA, moderate-vigorous-intensity physical activity.

Supplementary Table S9. Results from multilevel models of total sleep time predicting next day sedentary behavior and physical activity

|  | *β* Coefficients (SE) | | |
| --- | --- | --- | --- |
|  | SB | LPA | MVPA |
| Intercept | **81.00**  **(18.59)** | 8.79  (12.47) | 10.07  (10.00) |
| Age | -0.15  (0.21) | 0.15  (0.14) | 0.01  (0.12) |
| Sex  (Ref: Male) | -4.16  (2.91) | **5.41**  **(1.95)** | -1.25  (1.56) |
| BMI | 0.82  (1.16) | -0.44  (0.78) | -0.37  (0.63) |
| Smoking history  (Ref: Past/never) | **31.52**  **(10.87)** | **-24.26**  **(7.46)** | -7.22  (5.90) |
| Alcohol consumption  (Ref: Less than 1-6 time per week) | -1.27  (2.27) | 1.38  (1.52) | -0.13  (1.22) |
| Caffeine consumption  (Ref: Less than 1-6 time per week) | -4.60  (3.83) | **5.41**  **(2.58)** | -0.81  (2.06) |
| Hypertension  (Ref: No) | **5.25**  **(2.28)** | **-4.11**  **(1.53)** | -1.11  (1.23) |
| Hyperlipidemia  (Ref: No) | -4.62  (2.92) | **5.65**  **(1.96)** | -1.08  (1.57) |
| Depressive symptoms | -3.13  (1.99) | 2.41  (1.34) | 0.71  (1.07) |
| Total sleep time  (Between-person) | 0.00  (0.03) | -0.00  (0.02) | -0.00  (0.01) |
| Total sleep time  (Within-person) | -0.00  (0.01) | 0.00  (0.00) | 0.00  (0.00) |

Note: Bold numbers indicate *P* < .05; SE, standard error; BMI, body mass index; SB, sedentary behavior; LPA, low-intensity physical activity; MVPA, moderate-vigorous-intensity physical activity.

Supplementary Table S10. Results from multilevel models of sleep latency predicting next day sedentary behavior and physical activity

|  | *β* Coefficients (SE) | | |
| --- | --- | --- | --- |
|  | SB | LPA | MVPA |
| Intercept | **81.31**  **(18.58)** | 9.06  (12.46) | 9.48  (9.84) |
| Age | -0.16  (0.21) | 0.14  (0.14) | 0.01  (0.11) |
| Sex  (Ref: Male) | -4.15  (2.90) | **5.30**  **(1.94)** | -1.16  (1.53) |
| BMI | 0.92  (1.18) | -0.38  (0.79) | -0.53  (0.62) |
| Smoking history  (Ref: Past/never) | **32.55**  **(10.73)** | **-24.30**  **(7.37)** | -8.23  (5.75) |
| Alcohol consumption  (Ref: Less than 1-6 time per week) | -1.43  (2.27) | 1.30  (1.53) | 0.11  (1.20) |
| Caffeine consumption  (Ref: Less than 1-6 time per week) | -4.78  (3.84) | **5.36**  **(2.58)** | -0.57  (2.03) |
| Hypertension  (Ref: No) | **5.50**  **(2.27)** | **-4.06**  **(1.52)** | -1.40  (1.20) |
| Hyperlipidemia  (Ref: No) | -5.15  (2.85) | **5.56**  **(1.91)** | -0.44  (1.51) |
| Depressive symptoms | -3.17  (1.99) | 2.41  (1.33) | 0.76  (1.05) |
| Sleep latency  (Between-person) | 0.07  (0.10) | 0.01  (0.07) | -0.07  (0.05) |
| Sleep latency  (Within-person) | 0.01  (0.03) | -0.02  (0.02) | 0.01  (0.02) |

Note: Bold numbers indicate *P* < .05; SE, standard error; BMI, body mass index; SB, sedentary behavior; LPA, low-intensity physical activity; MVPA, moderate-vigorous-intensity physical activity.

Supplementary Table S11. Results from multilevel models of sleep efficiency predicting next day sedentary behavior and physical activity

|  | *β* Coefficients (SE) | | |
| --- | --- | --- | --- |
|  | SB | LPA | MVPA |
| Intercept | **79.32**  **(18.46)** | 9.51  (12.45) | 11.02  (9.84) |
| Age | -0.13  (0.22) | 0.14  (0.15) | -0.01  (9.84) |
| Sex  (Ref: Male) | -4.39  (2.94) | **5.21**  **(1.98)** | -0.83  (1.57) |
| BMI | 0.98  (1.16) | -0.46  (0.78) | -0.51  (0.62) |
| Smoking history  (Ref: Past/never) | **32.21**  **(10.66)** | **-24.39**  **(7.35)** | -7.81  (5.74) |
| Alcohol consumption  (Ref: Less than 1-6 time per week) | -1.20  (2.25) | 1.22  (1.52) | -0.03  (1.20) |
| Caffeine consumption  (Ref: Less than 1-6 time per week) | -4.24  (3.79) | **5.17**  **(2.56)** | -0.92  (2.02) |
| Hypertension  (Ref: No) | **5.29**  **(2.25)** | **-4.02**  **(1.52)** | -1.24  (1.20) |
| Hyperlipidemia  (Ref: No) | -5.16  (2.90) | **5.62**  **(1.95)** | -0.49  (1.55) |
| Depressive symptoms | -3.24  (1.98) | 2.43  (1.33) | 0.80  (1.05) |
| Sleep efficiency  (Between-person) | -0.11  (0.19) | 0.01  (0.13) | 0.10  (0.10) |
| Sleep efficiency  (Within-person) | -0.02  (0.05) | 0.02  (0.04) | 0.01  (0.03) |

Note: Bold numbers indicate *P* < .05; SE, standard error; BMI, body mass index; SB, sedentary behavior; LPA, low-intensity physical activity; MVPA, moderate-vigorous-intensity physical activity.

Supplementary Table S12. Results from multilevel models of wake after sleep onset predicting next day sedentary behavior and physical activity

|  | *β* Coefficients (SE) | | |
| --- | --- | --- | --- |
|  | SB | LPA | MVPA |
| Intercept | **80.85**  **(18.62)** | 8.80  (12.50) | 10.21  (10.00) |
| Age | -0.15  (0.22) | 0.14  (0.15) | 0.00  (0.12) |
| Sex  (Ref: Male) | -4.35  (2.99) | **5.42**  **(2.01)** | -1.07  (1.60) |
| BMI | 0.86  (1.17) | -0.45  (0.78) | -0.40  (0.63) |
| Smoking history  (Ref: Past/never) | **31.65**  **(10.71)** | **-24.40**  **(7.36)** | -7.23  (5.80) |
| Alcohol consumption  (Ref: Less than 1-6 time per week) | -1.37  (2.27) | 1.41  (1.52) | -0.05  (1.22) |
| Caffeine consumption  (Ref: Less than 1-6 time per week) | -4.55  (3.84) | **5.41**  **(2.58)** | -0.86  (2.06) |
| Hypertension  (Ref: No) | **5.38**  **(2.26)** | **-4.15**  **(1.52)** | -1.20  (1.21) |
| Hyperlipidemia  (Ref: No) | -5.02  (2.85) | **5.78**  **(1.91)** | -0.79  (1.53) |
| Depressive symptoms | -3.17  (2.00) | 2.42  (1.34) | 0.74  (1.07) |
| Wake after sleep onset  (Between-person) | 0.00  (0.04) | -0.00  (0.03) | -0.00  (0.02) |
| Wake after sleep onset  (Within-person) | -0.01  (0.01) | 0.00  (0.00) | 0.01  (0.01) |

Note: Bold numbers indicate *P* < .05; SE, standard error; BMI, body mass index; SB, sedentary behavior; LPA, low-intensity physical activity; MVPA, moderate-vigorous-intensity physical activity.

Supplementary Table S13. Results from multilevel models of REM latency predicting next day sedentary behavior and physical activity

|  | *β* Coefficients (SE) | | |
| --- | --- | --- | --- |
|  | SB | LPA | MVPA |
| Intercept | **80.42**  **(18.84)** | 7.36  (12.61) | 12.12  (10.02) |
| Age | -0.15  (0.22) | 0.17  (0.15) | -0.02  (0.12) |
| Sex  (Ref: Male) | -4.14  (2.90) | **5.33**  **(1.94)** | -1.19  (1.54) |
| BMI | 0.86  (1.18) | -0.37  (0.79) | -0.49  (0.63) |
| Smoking history  (Ref: Past/never) | **31.99**  **(10.73)** | **-23.99**  **(7.35)** | -7.98  (5.76) |
| Alcohol consumption  (Ref: Less than 1-6 time per week) | -1.39  (2.29) | 1.27  (1.53) | 0.11  (1.22) |
| Caffeine consumption  (Ref: Less than 1-6 time per week) | -4.64  (3.84) | **5.33**  **(2.58)** | -0.69  (2.04) |
| Hypertension  (Ref: No) | **5.30**  **(2.26)** | **-4.19**  **(1.51)** | -1.07  (1.20) |
| Hyperlipidemia  (Ref: No) | -4.76  (2.82) | **5.92**  **(1.89)** | -1.19  (1.50) |
| Depressive symptoms | -3.08  (2.01) | 2.52  (1.34) | 0.55  (1.07) |
| REM latency  (Between-person) | 0.01  (0.03) | 0.02  (0.02) | -0.03  (0.02) |
| REM latency  (Within-person) | -0.00  (0.01) | 0.01  (0.01) | -0.01  (0.01) |

Note: Bold numbers indicate *P* < .05; SE, standard error; BMI, body mass index; SB, sedentary behavior; LPA, low-intensity physical activity; MVPA, moderate-vigorous-intensity physical activity; REM, rapid eye movement sleep.

Supplementary Table S14. Results from multilevel models of N1 predicting next day sedentary behavior and physical activity

|  | *β* Coefficients (SE) | | |
| --- | --- | --- | --- |
|  | SB | LPA | MVPA |
| Intercept | **80.10**  **(18.68)** | 9.05  (12.54) | 10.68  (10.03) |
| Age | -0.15  (0.21) | 0.14  (0.14) | 0.01  (0.12) |
| Sex  (Ref: Male) | -3.90  (2.92) | **5.32**  **(1.96)** | -1.42  (1.57) |
| BMI | 0.80  (1.16) | -0.44  (0.78) | -0.36  (0.63) |
| Smoking history  (Ref: Past/never) | **31.30**  **(10.76)** | **-24.26**  **(7.39)** | -7.02  (5.83) |
| Alcohol consumption  (Ref: Less than 1-6 time per week) | -1.44  (2.28) | 1.44  (1.53) | -0.02  (1.22) |
| Caffeine consumption  (Ref: Less than 1-6 time per week) | -4.36  (3.86) | **5.34**  **(2.60)** | -0.98  (2.08) |
| Hypertension  (Ref: No) | **5.18**  **(2.28)** | **-4.10**  **(1.53)** | -1.05  (1.22) |
| Hyperlipidemia  (Ref: No) | -4.66  (2.82) | **5.70**  **(1.89)** | -1.07  (1.52) |
| Depressive symptoms | -3.06  (1.99) | 2.39  (1.34) | 0.66  (1.07) |
| N1  (Between-person) | -0.15  (0.29) | 0.05  (0.20) | 0.10  (0.16) |
| N1  (Within-person) | -0.03  (0.13) | 0.01  (0.10) | 0.01  (0.08) |

Note: Bold numbers indicate *P* < .05; SE, standard error; BMI, body mass index; SB, sedentary behavior; LPA, low-intensity physical activity; MVPA, moderate-vigorous-intensity physical activity; N, non-rapid eye movement sleep.

Supplementary Table S15. Results from multilevel models of N2 predicting next day sedentary behavior and physical activity

|  | *β* Coefficients (SE) | | |
| --- | --- | --- | --- |
|  | SB | LPA | MVPA |
| Intercept | **74.19**  **(18.95)** | 13.06  (12.74) | 12.61  (10.25) |
| Age | -0.06  (0.22) | 0.09  (0.15) | -0.02  (0.12) |
| Sex  (Ref: Male) | -4.16  (2.86) | **5.42**  **(1.92)** | -1.26  (1.55) |
| BMI | 0.89  (1.15) | -0.49  (0.77) | -0.40  (0.62) |
| Smoking history  (Ref: Past/never) | **32.40**  **(10.59)** | **-24.75**  **(7.29)** | -7.63  (5.77) |
| Alcohol consumption  (Ref: Less than 1-6 time per week) | -1.15  (2.24) | 1.30  (1.51) | -0.16  (1.21) |
| Caffeine consumption  (Ref: Less than 1-6 time per week) | -4.87  (3.79) | **5.59**  **(2.55)** | -0.71  (2.05) |
| Hypertension  (Ref: No) | **5.65**  **(2.24)** | **-4.34**  **(1.50)** | -1.28  (1.21) |
| Hyperlipidemia  (Ref: No) | -5.19  (2.78) | **5.97**  **(1.87)** | -0.81  (1.50) |
| Depressive symptoms | -3.02  (1.97) | 2.34  (1.32) | 0.67  (1.06) |
| N2  (Between-person) | 0.31  (0.17) | -0.19  (0.12) | -0.12  (0.09) |
| N2  (Within-person) | 0.09  (0.07) | -0.05  (0.06) | -0.04  (0.04) |

Note: Bold numbers indicate *P* < .05; SE, standard error; BMI, body mass index; SB, sedentary behavior; LPA, low-intensity physical activity; MVPA, moderate-vigorous-intensity physical activity; N, non-rapid eye movement sleep.

Supplementary Table S16. Results from multilevel models of N3 predicting next day sedentary behavior and physical activity

|  | *β* Coefficients (SE) | | |
| --- | --- | --- | --- |
|  | SB | LPA | MVPA |
| Intercept | **80.91**  **(18.32)** | 8.88  (12.29) | 10.08  (9.95) |
| Age | -0.13  (0.21) | 0.13  (0.14) | 0.00  (0.12) |
| Sex  (Ref: Male) | -4.84  (2.89) | **5.88**  **(1.94)** | -1.05  (1.57) |
| BMI | 0.83  (1.15) | -0.45  (0.77) | -0.37  (0.62) |
| Smoking history  (Ref: Past/never) | **34.01**  **(10.64)** | **-25.89**  **(7.31)** | -8.10  (5.82) |
| Alcohol consumption  (Ref: Less than 1-6 time per week) | -0.87  (2.25) | 1.09  (1.51) | -0.24  (1.22) |
| Caffeine consumption  (Ref: Less than 1-6 time per week) | -5.26  (3.80) | **5.86**  **(2.55)** | -0.60  (2.06) |
| Hypertension  (Ref: No) | **6.21**  **(2.28)** | **-4.74**  **(1.53)** | -1.44  (1.24) |
| Hyperlipidemia  (Ref: No) | **-5.55**  **(2.79)** | **6.24**  **(1.87)** | -0.72  (1.52) |
| Depressive symptoms | -3.19  (1.96) | 2.46  (1.32) | 0.73  (1.07) |
| N3  (Between-person) | -0.34  (0.18) | 0.23  (0.13) | 0.11  (0.10) |
| N3  (Within-person) | -0.10  (0.11) | 0.06  (0.08) | 0.03  (0.06) |

Note: Bold numbers indicate *P* < .05; SE, standard error; BMI, body mass index; SB, sedentary behavior; LPA, low-intensity physical activity; MVPA, moderate-vigorous-intensity physical activity; N, non-rapid eye movement sleep.

Supplementary Table S17. Results from multilevel models of sleepiness on rising predicting next day sedentary behavior and physical activity

|  | *β* Coefficients (SE) | | |
| --- | --- | --- | --- |
|  | SB | LPA | MVPA |
| Intercept | **84.81**  **(18.26)** | 5.77  (12.22) | 9.31  (9.74) |
| Age | -0.22  (0.21) | 0.21  (0.14) | 0.02  (0.11) |
| Sex  (Ref: Male) | -4.39  (2.84) | **5.45**  **(1.90)** | -1.07  (1.51) |
| BMI | 0.68  (1.13) | -0.31  (0.76) | -0.36  (0.60) |
| Smoking history  (Ref: Past/never) | **26.00**  **(10.56)** | **-19.00**  **(7.27)** | -6.97  (5.67) |
| Alcohol consumption  (Ref: Less than 1-6 time per week) | -1.06  (2.22) | 1.05  (1.48) | -0.00  (1.18) |
| Caffeine consumption  (Ref: Less than 1-6 time per week) | -3.03  (3.84) | 4.02  (2.57) | -0.98  (2.05) |
| Hypertension  (Ref: No) | **5.18**  **(2.21)** | **-4.17**  **(1.48)** | -0.99  (1.18) |
| Hyperlipidemia  (Ref: No) | -4.87  (2.74) | **5.81**  **(1.83)** | -0.94  (1.46) |
| Depressive symptoms | -2.90  (1.98) | 2.33  (1.33) | 0.55  (1.06) |
| Sleepiness on rising  (Between-person) | 0.11  (0.16) | -0.17  (0.11) | 0.06  (0.08) |
| Sleepiness on rising  (Within-person) | 0.01  (0.07) | -0.07  (0.05) | 0.06  (0.04) |

Note: Bold numbers indicate *P* < .05; SE, standard error; BMI, body mass index; SB, sedentary behavior; LPA, low-intensity physical activity; MVPA, moderate-vigorous-intensity physical activity.

Supplementary Table S18. Results from multilevel models of initiation and maintenance of sleep predicting next day sedentary behavior and physical activity

|  | *β* Coefficients (SE) | | |
| --- | --- | --- | --- |
|  | SB | LPA | MVPA |
| Intercept | **83.89**  **(17.99)** | 6.73  (12.03) | 9.27  (9.68) |
| Age | -0.21  (0.21) | 0.20  (0.14) | 0.02  (0.11) |
| Sex  (Ref: Male) | -4.28  (2.79) | **5.30**  **(1.87)** | -1.03  (1.50) |
| BMI | 0.79  (1.13) | -0.42  (0.75) | -0.37  (0.61) |
| Smoking history  (Ref: Past/never) | **24.99**  **(10.50)** | **-18.36**  **(7.22)** | -6.60  (5.68) |
| Alcohol consumption  (Ref: Less than 1-6 time per week) | -1.42  (2.21) | 1.33  (1.48) | 0.08  (1.19) |
| Caffeine consumption  (Ref: Less than 1-6 time per week) | -2.55  (3.75) | 3.81  (2.50) | -1.25  (2.02) |
| Hypertension  (Ref: No) | **5.26**  **(2.18)** | **-4.29**  **(1.46)** | -0.95  (1.17) |
| Hyperlipidemia  (Ref: No) | **-5.61**  **(2.72)** | **6.41**  **(1.82)** | -0.81  (1.47) |
| Depressive symptoms | -2.71  (1.95) | 2.15  (1.31) | 0.53  (1.05) |
| Initiation and maintenance of sleep  (Between-person) | 0.27  (0.15) | **-0.20**  **(0.10)** | -0.06  (0.08) |
| Initiation and maintenance of sleep  (Within-person) | 0.06  (0.04) | -0.04  (0.04) | -0.02  (0.03) |

Note: Bold numbers indicate *P* < .05; SE, standard error; BMI, body mass index; SB, sedentary behavior; LPA, low-intensity physical activity; MVPA, moderate-vigorous-intensity physical activity.

Supplementary Table S19. Results from multilevel models of frequent dreaming predicting next day sedentary behavior and physical activity

|  | *β* Coefficients (SE) | | |
| --- | --- | --- | --- |
|  | SB | LPA | MVPA |
| Intercept | **83.04**  **(18.15)** | 7.50  (12.12) | 9.33  (9.71) |
| Age | -0.20  (0.21) | 0.18  (0.14) | 0.02  (0.11) |
| Sex  (Ref: Male) | -3.83  (2.85) | **4.85**  **(1.90)** | -1.03  (1.52) |
| BMI | 0.85  (1.15) | -0.50  (0.77) | -0.33  (0.62) |
| Smoking history  (Ref: Past/never) | **27.47**  **(10.58)** | **-20.61**  **(7.27)** | -6.82  (5.70) |
| Alcohol consumption  (Ref: Less than 1-6 time per week) | -1.07  (2.22) | 1.08  (1.48) | -0.02  (1.19) |
| Caffeine consumption  (Ref: Less than 1-6 time per week) | -4.36  (3.78) | **5.45**  **(2.52)** | -1.08  (2.02) |
| Hypertension  (Ref: No) | **5.47**  **(2.20)** | **-4.49**  **(1.47)** | -0.96  (1.18) |
| Hyperlipidemia  (Ref: No) | -5.32  (2.74) | **6.26**  **(1.83)** | -0.95  (1.47) |
| Depressive symptoms | -2.78  (1.97) | 2.20  (1.32) | 0.56  (1.05) |
| Frequent dreaming  (Between-person) | 0.06  (0.18) | -0.11  (0.12) | 0.05  (0.10) |
| Frequent dreaming  (Within-person) | -0.07  (0.05) | 0.03  (0.04) | 0.03  (0.03) |

Note: Bold numbers indicate *P* < .05; SE, standard error; BMI, body mass index; SB, sedentary behavior; LPA, low-intensity physical activity; MVPA, moderate-vigorous-intensity physical activity.

Supplementary Table S20. Results from multilevel models of feeling refreshed predicting next day sedentary behavior and physical activity

|  | *β* Coefficients (SE) | | |
| --- | --- | --- | --- |
|  | SB | LPA | MVPA |
| Intercept | **83.59 (18.13)** | 6.99  (12.21) | 9.31  (9.65) |
| Age | -0.20  (0.21) | 0.19  (0.14) | 0.01  (0.11) |
| Sex  (Ref: Male) | -4.31  (2.82) | **5.27**  **(1.90)** | -0.97  (1.50) |
| BMI | 0.66  (1.13) | -0.32  (0.76) | -0.33  (0.60) |
| Smoking history  (Ref: Past/never) | **27.04**  **(10.53)** | **-19.83**  **(7.29)** | -7.17  (5.65) |
| Alcohol consumption  (Ref: Less than 1-6 time per week) | -1.13  (2.22) | 1.04  (1.50) | 0.08  (1.18) |
| Caffeine consumption  (Ref: Less than 1-6 time per week) | -3.61  (3.69) | 4.71  (2.49) | -1.09  (1.96) |
| Hypertension  (Ref: No) | **5.09**  **(2.21)** | **-4.25**  **(1.49)** | -0.83  (1.18) |
| Hyperlipidemia  (Ref: No) | -4.87  (2.73) | **5.89**  **(1.84)** | -1.04  (1.45) |
| Depressive symptoms | -2.99  (1.98) | 2.30  (1.34) | 0.67  (1.06) |
| Feeling refreshed  (Between-person) | 0.14  (0.15) | -0.07  (0.11) | -0.07  (0.08) |
| Feeling refreshed  (Within-person) | 0.02  (0.05) | -0.02  (0.04) | -0.01  (0.03) |

Note: Bold numbers indicate *P* < .05; SE, standard error; BMI, body mass index; SB, sedentary behavior; LPA, low-intensity physical activity; MVPA, moderate-vigorous-intensity physical activity.

Supplementary Table S21. Results from multilevel models of subjective global sleep quality predicting next day sedentary behavior and physical activity

|  | *β* Coefficients (SE) | | |
| --- | --- | --- | --- |
|  | SB | LPA | MVPA |
| Intercept | **83.99**  **(18.10)** | 6.55  (12.07) | 9.35  (9.70) |
| Age | -0.21  (0.21) | 0.20  (0.14) | 0.02  (0.11) |
| Sex  (Ref: Male) | -4.15  (2.81) | **5.21**  **(1.87)** | -1.07  (1.51) |
| BMI | 0.76  (1.13) | -0.40  (0.76) | -0.36  (0.61) |
| Smoking history  (Ref: Past/never) | **26.52**  **(10.49)** | **-19.55**  **(7.21)** | -6.94  (5.66) |
| Alcohol consumption  (Ref: Less than 1-6 time per week) | -1.23  (2.22) | 1.22  (1.48) | 0.00  (1.19) |
| Caffeine consumption  (Ref: Less than 1-6 time per week) | -3.25  (3.71) | 4.29  (2.47) | -1.02  (1.99) |
| Hypertension  (Ref: No) | **5.26**  **(2.19)** | **-4.26**  **(1.46)** | -0.98  (1.17) |
| Hyperlipidemia  (Ref: No) | -5.09  (2.71) | **6.01**  **(1.81)** | -0.94  (1.46) |
| Depressive symptoms | -3.02  (1.98) | 2.42  (1.32) | 0.58  (1.06) |
| OSA-MA total score  (Between-person) | 0.04  (0.04) | -0.05  (0.03) | 0.01  (0.02) |
| OSA-MA total score  (Within-person) | 0.00  (0.02) | -0.01  (0.01) | 0.01  (0.01) |

Note: Bold numbers indicate *P* < .05; SE, standard error; BMI, body mass index; SB, sedentary behavior; LPA, low-intensity physical activity; MVPA, moderate-vigorous-intensity physical activity; OSA-MA, Oguri-Shirakawa-Azumi sleep inventory, Middle-Aged and Aged version.

Supplementary Table S22. Characteristics of participants between included and excluded participants

|  |  | Included  participants  (n = 92) | | | Excluded participants  (n = 20) | | | Unpaired  *t* test or  chi square  *P* value |
| --- | --- | --- | --- | --- | --- | --- | --- | --- |
| **Variables** | | Mean | ± | SD | Mean | ± | SD |  |
| Age, years | | 73.9 | ± | 5.0 | 74.2 | ± | 5.6 | 0.852 |
| Female, n (%) | | 73 | (79.3) | | 18 | (90.0) | | 0.269 |
| Body mass index, kg/m^2^ | | 23.7 | ± | 3.2 | 23.6 | ± | 5.2 | 0.892 |
| Smoking history, n (%) | | 1 | (1.1) | | 0 |  | | 0.640 |
| Alcohol consumption (drinker), n (%) | | 35 | (38.0) | | 3 | (15.0) | | 0.049 |
| Caffeine consumption (drinker), n (%) | | 84 | (91.3) | | 17 | (85.0) | | 0.391 |
| **Medical history** | |  |  | |  |  |  |  |
| Hypertension, n (%) | | 35 | (38.0) | | 8 | (40.0) | | 0.870 |
| Hyperlipidemia, n (%) | | 18 | (19.6) | | 5 | (25.0) | | 0.586 |
| Diabetes, n (%) | | 4 | (4.3) | | 2 | (10.0) | | 0.309 |
| GDS score, points | | 3.2 | ± | 2.8 | 3.8 | ± | 2.4 | 0.405 |
| **Habitual physical activity** | |  |  |  |  |  |  |  |
| Wear time, hour | | 15.2 | ± | 1.4 | 14.9 | ± | 2.0 | 0.302 |
| SB, hour (% of wear time) | | 8.9 | (58.2) | | 8.5 | (57.6) | | 0.474 |
| LPA, hour (% of wear time) | | 5.3 | (34.7) | | 5.4 | (36.3) | | 0.720 |
| MVPA, hour (% of wear time) | | 1.1 | (7.1) | | 0.9 | (6.1) | | 0.391 |

Note: SD, standard deviation; GDS, geriatric depression scale; SB, sedentary behavior; LPA, low-intensity physical activity; MVPA, moderate-vigorous-intensity physical activity.

Supplementary Fig. S1. Comparison of midpoint of sleep time between weekday and weekend (n = 92)
